# Use of MALDI-TOF MS and culturomics to identify mosquitoes and their midgut microbiota

**DOI:** 10.1186/s13071-016-1776-y

**Published:** 2016-09-10

**Authors:** Fatalmoudou Tandina, Lionel Almeras, Abdoulaye K. Koné, Ogobara K. Doumbo, Didier Raoult, Philippe Parola

**Affiliations:** 1 Unité de Recherche en Maladies Infectieuses et Tropicales Emergentes (URMITE), UM63, CNRS 7278, IRD 198 (Dakar, Sénégal), Inserm 1095, Faculté de Médecine, Aix Marseille Université, 27 bd Jean Moulin, 13385 Marseille cedex 5, France; 2 Department of Epidemiology of Parasitic Diseases, Malaria Research and Training Center, University of Science, Techniques and Technologies of Bamako, Bamako, Mali; 3 Département d’Infectiologie de Terrain, Unité de Parasitologie, Institut de Recherche Biomédicale des Armées, Marseille, France

**Keywords:** *Anopheles gambiae* Giles, *Aedes albopictus*, *Culex quinquefasciatus*, Culturomics, MALDI-TOF MS, Microbiota

## Abstract

**Background:**

Mosquitoes transmit a wide range of human parasitic and viral diseases. In recent years, new techniques such as MALDI-TOF MS have been developed to identify mosquitoes at the species level, which is key for entomological surveys. Additionally, there is increasing interest in the mosquito microbiota and its role in vector capacity.

**Methods:**

The culturomics approach previously used in our laboratory to study human gut microbiota was applied to evaluate the midgut bacterial diversity of *Anopheles gambiae* (wild and laboratory strains), *Aedes albopictus* (wild and laboratory strains) and *Culex quinquefasciatus* (wild strains) in order to determine the influence of the environmental status on the midgut microbiota of the mosquitoes.

**Results:**

Mosquitoes collected in the field were accurately identified by MALDI-TOF MS analysis of their legs. Adult mosquito midgut microbiota was composed of four phyla, including Proteobacteria, Bacteroidetes, Actinobacteria and Firmicutes. The majority of the bacteria detected in the microbiota of mosquitoes were gram-negative and belong to the phylum Proteobacteria. MALDI-TOF MS identified for the first time a new bacterial species from *An. gambiae* midgut microbiota.

**Conclusion:**

In this study, the culturomics approach was found to be a reliable technique for exploring the diversity of the mosquito microbiota. MALDI-TOF MS was confirmed as a promising technique to identify mosquitoes collected in the field. Culturomics allowed the isolation of a new bacterial species not previously associated with mosquito vectors. The environment plays a role in the bacterial diversity of the microbiota, which could enable the development of new control strategies for mosquito-borne disease.

## Background

There are over 3500 different species of mosquitoes with a worldwide distribution [1]. The most described species that are able to transmit pathogens to humans and animals belong to the genera *Aedes*, *Culex* and *Anopheles* [1]. Mosquito vectors are not limited to tropical areas, where malaria, dengue and chikungunya are well-known threats for the local population and travellers [2, 3]. The tiger mosquito, *Aedes albopictus*, is an invasive species that has spread across the world in the last two decades [4]. The global expansion of *Ae. albopictus* may modify the worldwide epidemiology of arbovirus and increase the risk to humans of mosquito-borne diseases [5]. *Culex* spp. mosquitoes include vectors of human diseases such as arboviral diseases and lymphatic filariasis [1]. *Anopheles gambiae* (*s.l*.) mosquitoes are the major malarial vectors in sub-Saharan Africa [6]. Malaria parasites are transmitted from human to mosquito when a female *Anopheles* ingests a gametocyte-infected blood meal [6]. In the mosquito midgut, malaria parasites undergo a series of complex developmental stages and transmission depends on the success of the different transition steps [7].

Studies on the role of the microbiota within the gut of insects have increased in recent years [8, 9]. This has led to an intensification of studies focused on the microbiota of diverse mosquito species, including a potential influence on their vector competence [9, 10]. Some studies have shown the impact of the mosquito midgut microbiota in the defense against malaria parasites, with *Enterobacteriaceae* affecting the development of *P. falciparum* in the *An. gambiae* mosquito midgut [7]. Moreover, by using antibiotic treatments to clear the midgut microbiota, other studies have suggested a protective role of *An. gambiae* midgut bacteria against *Plasmodium* infections [11, 12]. It has also been suggested that antibiotics in ingested blood enhance the susceptibility of *An. gambiae* mosquitoes to malarial infection by disturbing their gut microbiota. In addition, antibiotic exposure increases mosquito survival and fecundity, which are factors increasing vectorial capacity [13].

In recent years, a new approach using special cultures, named culturomics, has been developed in our laboratory for the identification of not only the human gut microbiota, including common bacteria, but also minority bacterial populations [14]. Here, we used a culturomics approach to study the midgut bacterial diversity of three mosquito species, including wild and laboratory strains. The bacterial patterns of mosquito species reared in the laboratory were compared to mosquitoes collected in the field within their respective water sites to assess the effect of the environment on bacterial populations.

## Methods

### Laboratory-reared mosquitoes

*An. gambiae* Giles laboratory colonies and *Ae. albopictus* collected in the south of France in June 2013 were maintained by breeding at our laboratory in Marseille, France. Briefly, *Ae. albopictus* and *An. gambiae* from the laboratory were reared using standard methods at a temperature of 26 ± 1 °C, a relative humidity of 70–90 % and a photoperiod of 12 h (light/dark) in standalone incubators (Panasonic cooled incubator) [15]. After emerging from the pupae, the adults *An. gambiae* and *Ae. albopictus* were fed with a 10 % (w/v) sucrose solution. For egg production, blood meals were given through a parafilm membrane (Hemotek membrane feeding systems, Discovery Workshops, Accrington, England, UK) using fresh heparinized sheep blood over 1 h [5]. The blood-feeding was performed every three days, according to the gonotrophic cycle. Engorged female *An. gambiae* and *Ae. albopictus* were transferred into another cage and were maintained in standard conditions with 10 % (w/v) sucrose solution on cotton. Larvae were reared to the nymph stage in trays containing distilled water. Pupae were collected daily and transferred to mosquito cages (Bug Dorm 1, BioQuip Products, Gladwick Street, USA). Larvae were fed with fish food (TetraMin) until the pupal stage [16]. To explore the midgut microbiota of the laboratory mosquito colonies, the midguts of six specimens of *An. gambiae* and five specimens of *Ae. albopictus* were dissected.

Newly emerged adult *An. gambiae* specimens from our laboratory colonies were used in the present work. These anopheline mosquitoes were collected immediately after emergence and maintained under standard conditions and fed only with 10 % (w/v) sucrose solution for three days. Female mosquitoes were engorged on human blood (defibrinated human blood) over one hour. *Anopheles gambiae* females (three specimens) engorged on human blood were sacrificed 24 h after the blood meals (at day one) and midgut dissection was immediately performed under sterile conditions.

Additionally, one male and one female adult specimen from the F1 generation, resulting from *An. gambiae* female specimens who were fed on blood, were sacrificed 24 h after their emergence. There were a total of 12 *An. gambiae* specimens, including male (*n* = 3) and female (*n* = 3) specimens fed only on sucrose solutions, and females fed on human blood (*n* = 3). In addition, one male and one female adult specimen of the F1 generation of these last two groups were also tested. In this second experiment there was a sequential collection (i.e. D1, D3, D8 and F1) of mosquito midguts. Midguts were collected and analysed by culturomics.

### Mosquitoes in the wild

#### Collection of wild mosquitoes

*Aedes albopictus* was captured in Marseille by human landing catches in the garden of the Faculty of Medicine in August 2014. The collected *Ae. albopictus* mosquitoes were individually conserved in caps prior to transport to the laboratory. In Mali, 53 *An. gambiae* (*s.l*.) and 204 *Culex* spp. mosquitoes were captured using the CDC light trap from the Sikasso region (south Mali) from April to May, 2014. Specimens were sterilized in 70 % ethanol (2–10 min) and then rinsed in distilled water. Each adult mosquito was transferred individually to a 1.5 ml Eppendorf tube and the specimens were then kept at -80 °C and sent frozen to the URMITE laboratory (Marseille, France).

#### Identification of wild mosquitoes

The collected specimens were initially identified using morphological criteria [17]. Additionally, each specimen was submitted to MALDI-TOF MS for identification as previously described [18, 19]. Legs from each mosquito were homogenized manually in 20 μl of 70 % (v/v) formic acid and 20 μl of 50 % (v/v) acetonitrile in 1.5 ml microtubes using pellet pestles (Fischer Scientific, Strasbourg, France). The homogenates were centrifuged at 10,000 rpm for 20 s, and 1 μl of the supernatant of each sample was deposited on a steel target plate (Bruker DaltonicsTM, Wissembourg, France) into four spots for each sample [18]. Then, 1 μl of CHCA matrix composed of saturated a-cyano-4-hydroxycynnamic acid (SigmaH, Lyon. France), 50 % (v/v) acetonitrile, 2.5 % (v/v) trifluoroacetic acid and HPLC-grade water was directly overlaid on each sample on the target plate, dried for several minutes at room temperature and introduced into the MALDI-TOF MS instrument for analysis [19].

Protein mass profiles were obtained using Microflex LT MALDI-TOF Mass Spectrometry (Bruker Daltonics, Germany) with Flex Control software (Bruker Daltonics, Germany) as previously described [18, 20]. Measurements were performed in the linear positive-ion mode within a mass range of 2–20 kDa. Each spectrum corresponds to ions obtained from 240 laser shots performed in six regions of the same spot. The spectrum profiles obtained were visualized with flexAnalysis 3.3 software and exported to the MALDI Biotyper v. 3.0 (Bruker Daltonics, Germany) [19].

Molecular tools were also used to confirm the identification of some mosquitoes. DNA extractions from individual mosquito heads and thorax samples were performed with the EZ1 DNA Tissue Kit (Qiagen, Hilden, Germany) according to manufacturer recommendations. A set of primers specifically amplifying a fragment of the mosquito cytochrome *c* oxidase subunit I gene (mCOI) was used (LCO1490 (forward): 5'-GGT CAA CAA ATC ATA AGA TAT TGG-3'; HC02198 (reverse): 5'-TAA ACT TCA GGG TGA CCA AAA AAT CA-3') [21]. The PCR reaction contained 13 μl of sterile distilled water, 2.5 μl of 10X Phusion HF Buffer (15 mM), 2.5 μl of dNTPs (2 mM), 0.5 μl of each primer (10 μM), 0.25 μl of Hot star Taq (5units/μl), 1 μl of MgCl_2_ (25 mM) and 5 μl of extracted DNA. Reactions were amplified through 35 cycles at the following parameters: 10 min at 95 °C, 1 min at 95 °C, 1 min at 40 °C, 1.5 min at 72 °C, followed by a final extension step at 72 °C for 7 min.

A set of primers specifically amplifying a fragment of 310 bp of the *An. gambiae* mosquito complex > Acomplex_28S_MBF AGC KCG TCT TGG TCT GGG G and > Acomplex_28S_MBR GCC GAC AAG CTC AYT AGT GT were designed in the URMITE laboratory based on the publication of Fanello et al., and PCR reactions were processed as described [22]. Positive PCR products were then purified and sequenced using the same respective primers with the BigDye version 1–1 Cycle Sequencing Ready Reaction Mix (Applied Biosystems, Foster City, CA) and an ABI 3100 automated sequencer (Applied Biosystems). The sequences were assembled and analyzed using the ChromasPro software (version 1.34) (Technelysium Pty. Ltd., Tewantin, Australia) and BLAST website (http://blast.ncbi.nlm.nih.gov).

### Water from breeding sites

For mosquito colonies reared in the laboratory (i.e. *An. gambiae* and *Ae. albopictus*), 200 μl of the laboratory breeding water was collected with a Pasteur pipette and put into the 1.5 ml Eppendorf sterile tubes for culturomics analyses. Breeding water was then used for the culturomics experiments to control and compare the bacterial diversity between the environmental breeding site and the adult mosquito midgut.

The breeding water of the wild mosquitoes from Marseille was recovered with a ladle near the place where the mosquitoes had been collected on humans. The water was then transferred to a 15 ml sterile tube and transported to the laboratory. In the Sikasso region, in Mali, three breeding water sites were selected and collected with ladle sampling, prior to being transferred to a 15 ml sterile tube and transported to the laboratory. Then, the samples were stored at -80 °C until they were transported frozen to URMITE. Each breeding site was geo-positioned as follows: breeding site 1 (-5°66'13.1"N, 11°30'95.2"E); breeding site 2 (-5°60'80.6"N, 11°30'30.6"E) and breeding site 3 (-5°60'73.8"N, 11°30'58.0"E).

### Mosquito gut dissection

Adult mosquitoes were anesthetized with cold at -20 °C for 10 min. All midgut mosquitoes from laboratory colonies (*An. gambiae* and *Ae. albopictus*) and wild mosquitoes were dissected under sterile conditions. Mosquitoes were surface-sterilized in 70 % (v/v) ethanol for 2–10 min, then rinsed three times in a sterile saline buffer 0.9 % (w/v) NaCl (Laboratoires Gilbert, France). The midgut was carefully removed under a stereo microscope (10× magnification) using clean forceps.

Midguts from the *An. gambiae* laboratory colony, *Ae. albopictus* laboratory colony and *Ae. albopictus* wild colony were placed individually and midguts from the *An. gambiae* wild colony and *C. quinquefasciatus* wild colony were pooled in sterile Eppendorf tubes containing 200 μl of 0.9 % (w/v) NaCl (Laboratoires Gilbert, France) and homogenized using a single-use pestle and centrifuge and vortex [23, 24]. There were a total of six *An. gambiae* from the laboratory in Marseille for the first experiment and 12 *An. gambiae* from the laboratory in Marseille for the second experiment; five *Ae. albopictus* from the laboratory in Marseille, four mosquitoes field-collected in Marseille and twelve mosquitoes field-collected in Mali.

### Culturomics procedure

The standard and optimal conditions for the culturomics approach were used, based on previous work performed in our laboratory, notably for research on the human gut microbiota [14]. This technique started with pre-incubation: a special liquid media comprising 15 g/l brain heart infusion (Becton, Dickinson and Company, Sparks, MD 21152 USA; 38800 Le Pont-de-Claix, France), 5 g/l Bacto yeast extract (Becton, Dickinson and Company, Sparks, MD 21152 USA; 38800 Le Pont-de-Claix, France), 5 g/l proteose peptone (Oxoid Ltd, Basingstoke, Hampshire, England), 1000 ml of sterile water (Fresenius Kabi France, 5 Place de Marivel, 92310 Sèvres, France) and 5 % (v/v) sheep blood in aerobic and anaerobic conditions at 28 °C for 1 month. We inoculated them on 5 % (v/v) sheep blood agar (bioMérieux, Marcy l’Etoile, France) after performing ten serial dilutions from 1/10 to 1/10^-10^, allowing the growth of fastidious bacteria in order to isolate a maximum of bacterial species. This operation was carried out every 5 days from day 1 to day 25 (i.e. D1, D5, D10, D15, D20 and D25). Bacterial colonies were then isolated on 5 % (v/v) sheep blood agar after 24 h, and submitted to mass spectrometry (MALDI-TOF MS) for identification. Bacteria not identified by MALDI-TOF MS were then submitted to molecular biology for taxonomic determination by 16S sequencing.

### Bacterial identification

#### Mass spectrometry (MALDI-TOF)

Each bacterial colony obtained from culture was deposed in duplicate directly onto a MALDI-TOF plate target (Bruker DaltonicsTM, Wissembourg, France) and covered with 1.5 μl of the matrix solution composed of a saturated solution of α-cyano-4-hydroxycinnamic acid (SigmaH, Lyon, France) diluted in 500 μl of acetonitrile 50 % (v/v), 250 μl of trifluoroacetic acid 10 % (v/v) and 250 μl of HPLC water and dried for several minutes at room temperature. The target plate was then submitted to MALDI-TOF mass spectrometry for bacterial identification as previously described [25]. To control loading on mass spectra steel, matrix quality and MALDI-TOF apparatus performance, the matrix solution was loaded in duplicate onto each MALDI-TOF plate with and without a Bacterial Test Standard (Bruker Protein Calibration Standard I). A Microflex LT MALDI-TOF mass spectrometer (Bruker Daltonics, Germany) was used for bacterial identification according to the manufacturer’s recommendations. Spectra were recorded in a linear mode, within a mass range of 2,000 to 20,000 Daltons (Da). For each spectrum, data for multiple laser shots were collected, summed and analysed. A maximum of 100 peaks was used for each spectrum, and these peaks were compared with the computer database at the Bruker base and the lab-specific base at La Timone hospital. An isolate was considered to be correctly and significantly identified at the species level when the queried spectrum had a log score value (LSV) ≥ 1.9 [26]. Every unidentified colony was tested successively three times. When the strain remained unidentified, the 16S rRNA gene was sequenced. Spectra from new bacteria species not yet included in the database and identified by 16S rRNA sequencing were added to the database.

### 16S rRNA gene sequencing

Identification with 16S rRNA gene sequencing was performed for the bacteria not identified by MALDI-TOF MS. For this, the bacterial strain was suspended in 200 μl of sterile water and was heated at 100 °C for 10 min. The 16S rRNA gene was amplified by PCR using the universal primer pair fd1 and rp2 and an annealing temperature of 52 °C. The PCR products were purified using a NucleoFast 96 PCR kit (Nanogen, San Diego, USA). The sequence reactions were performed with the BigDye Terminator v1.1 Cycle Sequencing Kit (Perkin-Elmer), with primers fd1, rp2, 536 F, 536R, 800 F, 800R, 1050 F and 1050R (Table 1). The products of the sequencing reaction were purified, and the sequences were analysed using an ABI PRISM 3130xl Genetic Analyzer (Applied Biosystems). The obtained sequences were compared with the GenBank database using BLAST software. A threshold similarity value of > 98.7 % was chosen for identification at the species level [27]. Below this value, a new species was suspected, and the isolated strain was characterized in detail using phenotypic analyses and electron microscopy and genome sequencing.

**Table 1:** List of primers used for 16S rRNA amplification and sequencing

Primers names	Primer sequences (5′–3′)	Temperature (°C)
fd1	AGA GTT TGA TCC TGG CTC AG	52
rp2	ACG GCT ACC TTG TTA CGA CTT	52
536 F	CAG CAG CCG CGG TAA TAC	50
536R	GTA TTA CCG CGG CTG CTG	50
800 F	ATT AGA TAC CCT GGT AG	50
800R	CTA CCA GGG TAT CTA AT	50
1050 F	TGT CGT CAG CTC GTG	50
1050R	CAC GAG CTG ACG ACA	50

## Results

### Identification of the mosquitoes collected in the field

Four mosquitoes were collected using the human landing catches method in the Timone hospital garden (Marseille, France). They were morphologically identified as *Ae. albopictus* specimens. The submission of their legs for MALDI-TOF MS analysis confirmed that the four specimens were *Ae. albopictus* (LSVs > 1.9).

In Sikasso, Mali, 257 were mosquitoes captured with the CDC light trap, 53 and 204 were identified by morphologic keys as *An. gambiae* (*s.l*.) and 204 *Culex* spp. mosquitoes, respectively. Legs of 6 *An. gambiae* (*s.l.*) and 6 *Culex* spp. specimens were submitted to MALDI-TOF MS for identification. MALDI-TOF MS results confirmed the morphological identification of *An. gambiae* (*s.l*.) (LSVs > 1.9) and revealed that *Culex* spp. specimens were all *C. quinquefasciatus* (LSVs > 1.9). Among these field-collected mosquitoes in Mali, six *An. gambiae* and six *C. quinquefasciatus* were selected for analysis of their microbiota.

### Mosquitoes reared in the laboratory and their respective breeding water

In the first experiment, the midgut microbiota of six *An. gambiae* from the laboratory colony were analysed by culturomics. A wide variety of bacteria were found at the end of the sequential 1-month culture. A total of ten distinct bacterial species were identified (Table 2). Bacterial diversity included three phyla: Proteobacteria (50 %), Firmicutes (30 %) and Bacteroidetes (20 %). Two bacterial species, *Cedecea lapagei* and *Serratia marcescens*, were isolated in five midguts (83 %) among the six tested. *Enterococcus faecium*, *Elizabethkingia meningoseptica* and *Serratia ureilytica* were isolated in four midguts (67 %). *Elizabethkingia miricola*, *Enterobacter cloacae*, *Enterococcus avium*, *Enterococcus raffinosus* and *Enterobacter kobei* were less frequently detected in the six midguts of the *An. gambiae* tested (Table 2).

**Table 2:** List of bacteria identified in the midguts of *Anopheles gambiae* bred under laboratory conditions

Bacteria	Midgut 1	Midgut 2	Midgut 3	Midgut 4	Midgut 5	Midgut 6
*Cedecea lapagei*		×	×	×	×	×
Enterococcus faecium*	×	×		×	×	
Elizabethkingia miricola*	×		×		×	
*Elizabethkingia meningoseptica*	×		×		×	×
*Enterobacter cloacae*		×				×
***Serratia marcescens***		×	×	×	×	×
Enterococcus avium*			×			
Enterococcus raffinosus*			×	×	×	
Serratia ureilytica*		×	×	×	×	
Enterobacter kobei*		×				×

Bacterial species reported for the first time in *Anopheles gambiae* are indicated by an asterisk. Presence of bacteria is indicated by ×. Bold corresponds to bacterial species common in the midgut and breeding water

In a second experiment, 12 *An. gambiae* specimens of the laboratory colony were used. A total of 16 distinct bacterial species were identified belonging to 12 genera: *Enterococcus*, *Enterobacter*, *Serratia*, *Acinetobacter*, *Elizabethkingia*, *Microbacterium*, *Staphylococcus*, *Streptococcus*, *Cedecea*, *Pseudomonas*, *Rhodococcus* and *Klebsiella* in *An. gambiae* mosquito midguts (Table 3). This diversity is composed of the bacteria of four phyla: Proteobacteria (60 %), Firmicutes (20 %), Bacteroidetes (6.67 %) and Actinobacteria (13.33 %) (Table 3). A total of six bacterial species were common to *An. gambiae* midgut and breeding water, and seven bacterial species were found only in the breeding water (Table 3).

**Table 3:** List of bacteria identified in the midguts of laboratory-bred *Anopheles gambiae* non-engorged or engorged and in breeding water

Bacteria	Female fed on blood	Female sj	Male	Breeding water
*Aeromonas hydrophila*				×
*Aeromonas jandaei*				×
***Klebsiella oxytoca***			×	×
***Enterococcus faecium***				×
Enterobacter asburiae*		×	×	×
Enterobacter kobei*	×	×	×	×
***Serratia marcescens***	×	×	×	×
*Serratia fonticola*				×
Serratia ureilytica*	×			×
*Serratia plymuthica*				×
*Bacillus cereus*				×
*Sphingobacterium multivorum*				×
*Oceanobacillus massiliensis*				×
Acinetobacter baylyi*			×	
*Elizabethkingia meningoseptica*	×	×	×	
Microbacterium maritypicum*		×		
Staphylococcus epidermidis*		×		
Streptococcus sanguinis*		×		
Streptococcus mitis*	×			
*Cedecea lapagei*	×		×	
Pseudomonas gessardii*	×			
Rhodococcus erythropolis*	×			
*Enterobacter cloacae*	×	×	×	

Bacterial species reported for the first time in *Anopheles gambiae* are indicated by an asterisk. Presence of bacteria is indicated by ×. Bold corresponds to bacterial species common in the midgut and breeding water

*Abbreviations*: sj, sample from a female fed only on sweet juice, corresponding to the negative control

**Fig. 1:**
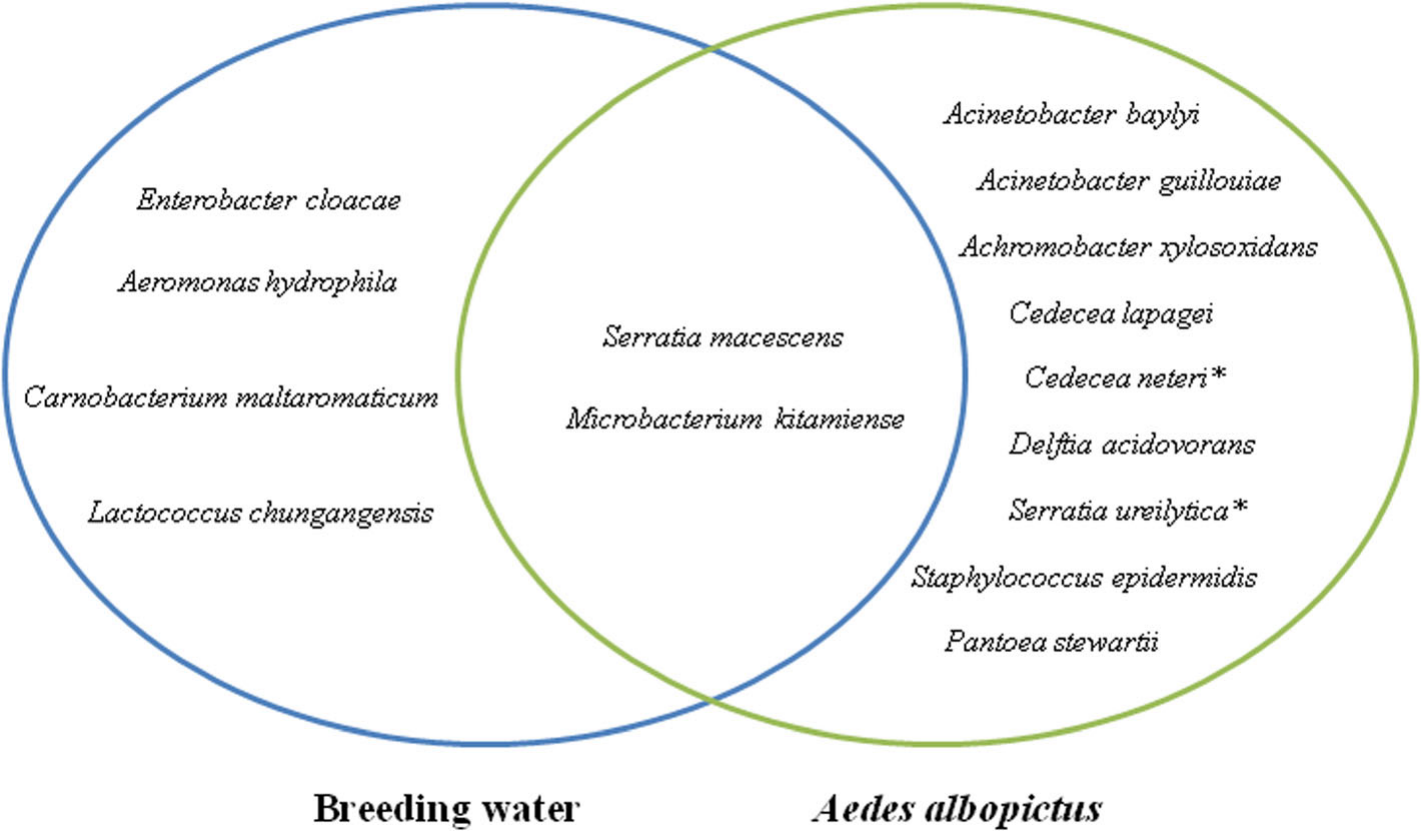
Isolation and identification of bacteria in the breeding water and midgut of an *Aedes albopictus* laboratory colony (Marseille, France). Bacterial species reported for the first time in *Aedes albopictus* microbiota are indicated by an asterisk (*)

A wide variety of bacteria in the midgut of *Ae. albopictus* from the laboratory was observed. A total of 11 distinct bacterial species were identified in mosquito midguts (Fig. 1). *Acinetobacter baylyi* were isolated in three midguts (60 %). *Acinetobacter guillouiae*, *Achromobacter xylosoxidans*, *Cedecea lapagei*, *Cedecea neteri*, *Serratia marcescens*, *Serratia ureilytica*, *Staphylococcus epidermidis*, *Pantoea stewartii* and *Microbacterium kitamiense* were less frequently represented (Fig. 1). The breeding water of *Ae. albopictus* was analysed, and five bacterial species were identified (Fig. 1). Among them, *Serratia marcescens* and *Microbacterium kitamiense* were found both in the *Ae. albopictus* mosquito midguts and their breeding water. Three genera, *Aeromonas*, *Carnobacterium* and *Lactobacillus*, were found only in the breeding water (Fig. 1).

### Mosquitoes collected in the field and their breeding water

**Fig. 2:**
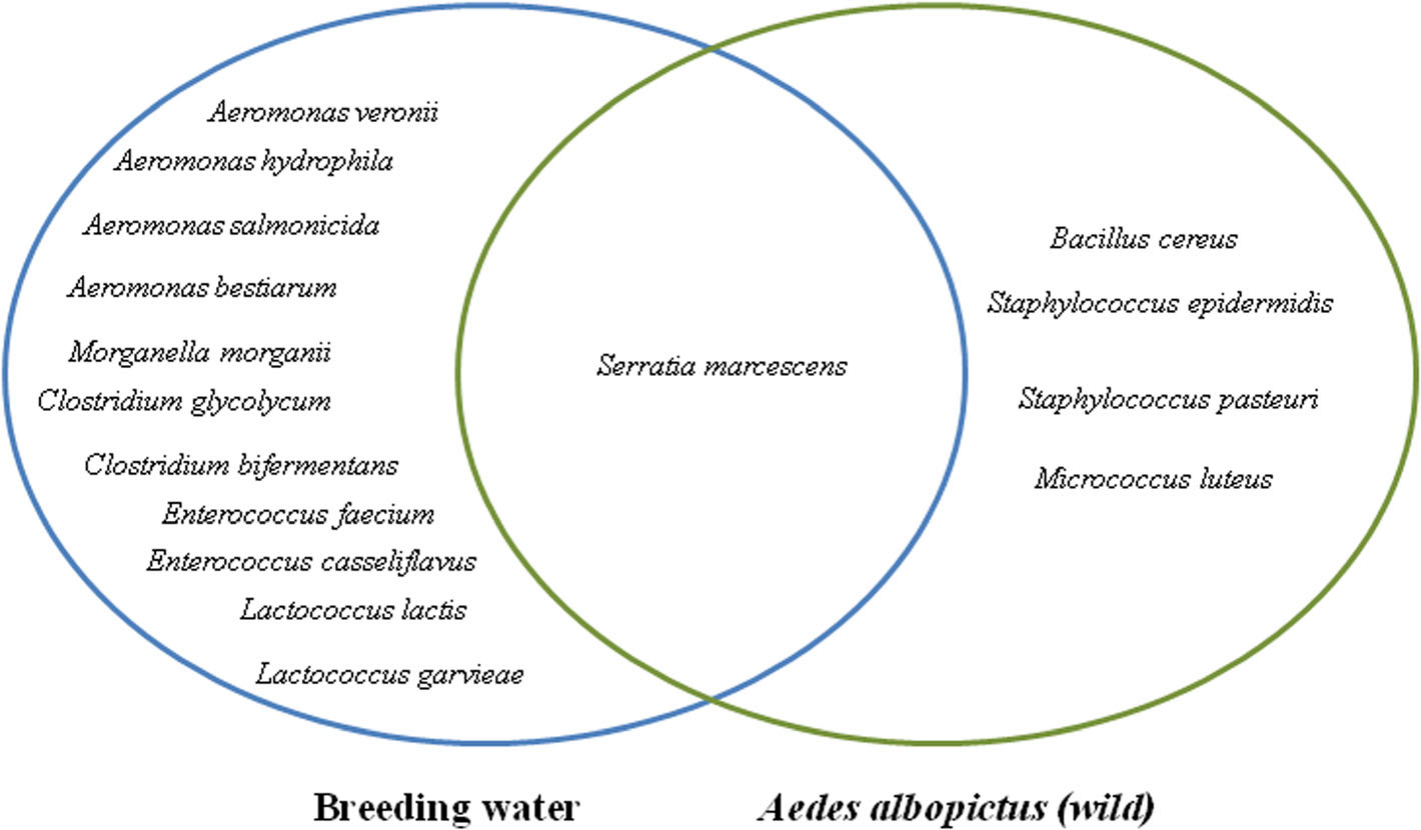
Isolation and identification of bacteria in the breeding water and midgut of *Aedes albopictus* wild colonies (Marseille, France)

Five distinct bacterial species were isolated from the midguts of *Ae. albopictus* field-collected mosquitoes (Marseille), including four genera: *Bacillus*, *Micrococcus*, *Staphylococcus* and *Serratia* (Fig. 2).

*Serratia marcescens* bacteria were common in the *Ae. albopictus* mosquito midguts and their breeding water. Eleven bacterial species from five genera, *Aeromonas*, *Clostridium*, *Enterococcus*, *Lactococcus* and *Morganella*, were found only in their breeding water.

**Fig. 3:**
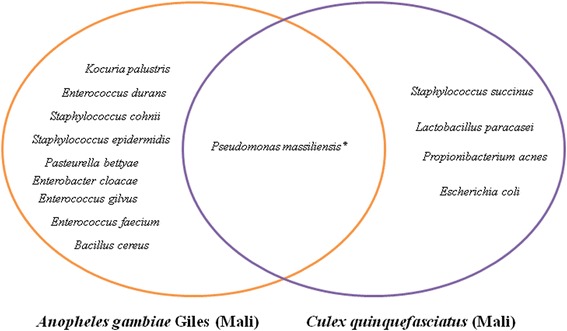
Isolation and identification of bacteria in the midgut of *Anopheles gambiae* and *Culex quinquefasciatus* wild colonies in Sikasso (Mali). Bacterial species reported for the first time in *Anopheles gambiae* and *Culex quinquefasciatus* microbiota are indicated by an asterisk (*)

The gut microbiota of *An. gambiae* in the wild (Mali) was composed of ten bacterial species from seven genera: *Enterobacter*, *Pasteurella*, *Pseudomonas*, *Bacillus*, *Enterococcus*, *Staphylococcus* and *Kocuria* (Fig. 3).

Midgut microbiota of *C. quinquefasciatus* from Mali was composed of five bacterial species from five genera: *Pseudomonas*, *Escherichia*, *Propionibacterium*, *Staphylococcus* and *Lactobacillus* (Fig. 3).

In breeding water sites, 51 bacterial species were cultivated representing 15 genera: *Acinetobacter, Aeromonas*, *Arthrobacter*, *Bacillus*, *Clostridium*, *Delftia*, *Enterococcus*, *Lactococcus*, *Lysinibacillus*, *Pseudomonas*, *Raoultella*, *Robinsonella*, *Rothia*, *Shewanella* and *Serratia*. Two bacterial species, *Bacillus cereus* and *Enterococcus faecium*, were common to the *An. gambiae* wild strains and breeding water (Fig. 3, Table 4).

**Table 4:** Composition of the microbiota of three breeding water samples in Sikasso (Mali)

Bacteria	Breeding water 1	Breeding water 2	Breeding water 3
*Acinetobacter lwoffii*	×		×
*Acinetobacter towneri*	×		
*Aeromonas hydrophila*	×		
*Aeromonas veronii*	×		
*Arthrobacter gandavensis*		×	
***Bacillus cereus***	×	×	×
*Bacillus idriensis*	×		
*Bacillus megaterium*			×
*Clostridium absonum*			×
*Clostridium amylolyticum*			×
*Clostridium anorexicamassiliense*	×	×	×
*Clostridium aerotolerans*	×		
*Clostridium butyricum*		×	×
*Clostridium bifermentans*	×	×	×
*Clostridium cadaveris*			×
*Clostridium celerecrescens*	×	×	
*Clostridium collagenovorans*			×
*Clostridium glycolycum*	×		×
*Clostridium ihumii*			×
*Clostridium lituseburense*	×	×	×
*Clostridium n*	×		×
*Clostridium perfringens*		×	
*Clostridium sardiniense*			×
*Clostridium sartagoforme*	×		
*Clostridium senegalensis*		×	
*Clostridium sordellii*		×	
*Clostridium sphenoides*		×	
*Clostridium sporogenes*	×		
*Clostridium tertium*	×		×
*Delftia acidovorans*		×	
*Enterococcus casseliflavus*	×	×	×
***Enterococcus faecium***	×	×	×
*Enterococcus hirae*		×	×
*Enterococcus italicus*			×
*Enterococcus mundtii*		×	
*Enterococcus termitis*			
*Lactococcus chungangensis*	×	×	×
*Lysinibacillus sphaericus*		×	
*Pseudomonas chlororaphis*		×	
*Pseudomonas corrugata*		×	
*Pseudomonas fluorescens*	×		
*Pseudomonas jessenii*		×	
*Pseudomonas monteilii*	×		
*Pseudomonas putida*	×	×	
*Pseudomonas rhodesiae*	×		
*Pseudomonas tolaasii*	×		
*Raoultella ornithinolytica*		×	
*Robinsoniella peoriensis*		×	
*Rothia aeria*	×		
*Shewanella profunda*	×		
*Serratia fonticola*		×	×

Presence of bacteria is indicated by ×

Bold corresponds to bacterial species common in the midgut and breeding water

## Discussion

This work analysed the midgut microbiota composition of mosquitoes reared in the laboratory and collected in the wild and compared this midgut microbiota diversity with their respective breeding sites, using an original strategy based on a special culture, the culturomics technique.

Furthermore, for the definitive identification of mosquitoes collected in the field, a new innovative method based on the analysis of mosquito leg protein spectra obtained by MALDI-TOF MS was used [19, 28].

This approach has been applied here for the first time to mosquitoes collected in the field from Africa. This is further evidence that use of MALDI-TOF MS to identify mosquitoes is a rapid, accurate analysis technique, at low cost in terms of consumables [19, 28].

Several previous studies have already analysed mosquito microbiota and the water of their respective breeding sites [10]. Most of these studies used molecular approaches, mainly based on analysing sequences of the 16S ribosomal RNA gene and cultures of mosquito midgut microbiota [10, 29].

The culturomics approach used in this work revealed a wide diversity of the midgut microbiota of *An. gambiae* (wild and laboratory strains*), Ae. albopictus* (wild and laboratory strains) and *C. quinquefasciatus* (wild strains).

The majority of the bacteria detected in the microbiota of mosquitoes were gram-negative and belong to the phylum Proteobacteria, similar to other studies [10, 29]. However, 17 new bacterial species not previously identified in *An gambiae* midgut microbiota have been isolated here: *Serratia ureilytica*, *Enterobacter kobei*, *Enterococcus faecium*, *Enterococcus avium*, *Enterococcus raffinosus*, *Elizabethkingia miricola*, *Acinetobacter baylyi*, *Cedecea neteri*, *Enterobacter asburiae*, *Pseudomonas gessardii*, *Streptococcus sanguinis*, *Streptococcus mitis*, *Staphylococcus epidermidis*, *Clostridium perfringens*, *Microbacterium maritypicum*, *Pseudomonas massiliensis* and *Rhodococcus erythropolis.*

Interestingly, among the bacterial colonies submitted to MALDI-TOF MS identification, one isolated in breeding water (Mali) was not identified, and corresponded to *Lactococcus chungangensis* according to 16S sequencing. This bacterial species was then implemented in the MALDI-TOF MS.

Moreover, a bacterial species, *Pseudomonas massiliensis*, recently isolated in the Timone laboratory, which is under description (D. Raoult, personal communication), was isolated in the *An. gambiae* and *C. quinquefasciatus* midgut and their breeding water collected from Mali.

Six bacterial species were commonly found in the midgut of *An. gambiae* laboratory colonies from Marseille and its respective breeding water. Moreover, 12 and seven bacterial species were found only in the midgut of *An. gambiae* laboratory colonies from Marseille and its breeding water, respectively. Three bacterial species (*Enterococcus faecium, Enterobacter cloacae* and *Staphylococcus epidermidis*) were commonly found both in the midgut of *An. gambiae* wild strains and laboratory strains.

The gut microbiota of *An. gambiae* in the wild (Mali) was composed of seven genera: *Enterobacter*, *Pasteurella*, *Pseudomonas*, *Bacillus*, *Enterococcus*, *Staphylococcus* and *Kocuria.* The gut microbiota of *An. gambiae* from field collection (Cameroon) was found to be dominated by *Comamonas*, *Serratia*, *Pseudomonas*, *Burkholderia* and *Brevundimonas* bacteria by pyrosequencing analysis [30].

We found that some bacterial species were common in the midgut of *An. gambiae* and *Ae. albopictus* laboratory strains*.*

Comparing the *Ae. albopictus* laboratory strain with those of the *Ae. albopictus* wild strain, we observed that *Serratia marcescens* was the only bacterial species found in common. Eight bacterial species were only found in the midgut of *Ae. albopictus* in the laboratory. Conversely, four were specific for the midguts of the *Ae. albopictus* wild strain. A difference was observed between the midgut microbiota of *Ae. albopictus* laboratory and wild strains. In addition to the contribution to the knowledge of bacterial species associated with the microbiota of mosquito vectors, these results suggest that the environment plays a major role in variations of the midgut microbiota diversity of mosquitoes. All the bacteria isolated from the laboratory and wild mosquito microbiota and breeding water are ubiquitous in the environment and are found in water and soil, as well in association with plants, insects, humans and other animals [31–33]. These results correlate with other studies; namely that the environmental conditions of the vectors are key determinants in shaping the midgut microbiota [24].

The main limitation of our growth conditions is that culturomics does not allow the growth of some strictly anaerobic bacteria [14]. Strategies are currently under development by the team culturomics to enable the growth of these bacteria considered uncultured by culturomics [34].

The sample size of laboratory mosquitoes used in this study is higher than the number of wild mosquitoes collected; this may explain the increase in the number of bacteria isolated in the laboratory mosquitoes.

Despite the previous studies of the mosquito midgut microbiota, it is still necessary to extend our knowledge in this domain by using new tools for exploration, such as culturomics. This culturomics approach allowed the isolation of bacterial species not previously associated with these vectors, and will aid the development of new control strategies for mosquito-borne diseases.

## Conclusions

To conclude, diverse bacterial species were found in common in the midguts of adult *An. gambiae*, *Ae. albopictus* and *C. quinquefasciatus* and in breeding water. The majority of the bacterial species belong to the phyla Proteobacteria and Firmicutes. Culturomics allowed the isolation of bacterial species identified for the first time in *An. gambiae* midgut. This study demonstrates a wide diversity of new species of bacteria associated with the mosquito microbiota, which may be targets for vector control strategies. Our study shows that the immediate environment plays an important role in the acquisition of bacteria by the mosquito. The innovative culturomics technique and MALDI-TOF MS application are evidence of the growth and correct identification of bacteria, isolated without ambiguity from the mosquito microbiota.

## Abbreviations

16S rRNA: 16S ribosomal ribonucleic acid
B: *Bacillus*
C: *Culex*
HPLC: High-performance liquid chromatography
LSV: Log score value
MALDI-TOF MS: Matrix-Assisted Laser Desorption Ionization Time-Of-Flight Mass Spectrometry
MRTC: Malaria Research and Training Center
URMITE: Unité de Recherche sur les Maladies Infectieuses et Tropicales Emergentes
